# Identification of Targeted Proteins by Jamu Formulas for Different Efficacies Using Machine Learning Approach

**DOI:** 10.3390/life11080866

**Published:** 2021-08-23

**Authors:** Sony Hartono Wijaya, Farit Mochamad Afendi, Irmanida Batubara, Ming Huang, Naoaki Ono, Shigehiko Kanaya, Md. Altaf-Ul-Amin

**Affiliations:** 1 Department of Computer Science, IPB University, Kampus IPB Dramaga Wing 20 Level 5, Bogor 16680, Indonesia; 2 Tropical Biopharmaca Research Center, IPB University, Kampus IPB Taman Kencana, Bogor 16128, Indonesia; fmafendi@apps.ipb.ac.id (F.M.A.); ime@apps.ipb.ac.id (I.B.); 3 Department of Statistics, IPB University, Kampus IPB Dramaga Wing 22 Level 4, Bogor 16680, Indonesia; 4 Department of Chemistry, IPB University, Kampus IPB Dramaga Wing 1 Level 3, Bogor 16128, Indonesia; 5 Computational Systems Biology Laboratory, Graduate School of Science and Technology, Nara Institute of Science and Technology, 8916-5 Takayama, Ikoma 630-0192, Nara, Japan; alex-mhuang@is.naist.jp (M.H.); nono@is.naist.jp (N.O.); skanaya@gtc.naist.jp (S.K.)

**Keywords:** compound–protein interaction, Jamu, machine learning, drug discovery, herbal medicine

## Abstract

Background: We performed in silico prediction of the interactions between compounds of Jamu herbs and human proteins by utilizing data-intensive science and machine learning methods. Verifying the proteins that are targeted by compounds of natural herbs will be helpful to select natural herb-based drug candidates. Methods: Initially, data related to compounds, target proteins, and interactions between them were collected from open access databases. Compounds are represented by molecular fingerprints, whereas amino acid sequences are represented by numerical protein descriptors. Then, prediction models that predict the interactions between compounds and target proteins were constructed using support vector machine and random forest. Results: A random forest model constructed based on MACCS fingerprint and amino acid composition obtained the highest accuracy. We used the best model to predict target proteins for 94 important Jamu compounds and assessed the results by supporting evidence from published literature and other sources. There are 27 compounds that can be validated by professional doctors, and those compounds belong to seven efficacy groups. Conclusion: By comparing the efficacy of predicted compounds and the relations of the targeted proteins with diseases, we found that some compounds might be considered as drug candidates.

## 1. Introduction

Identification of compounds derived from herbal medicines and natural products has shown potential in drug discovery and drug development [1,2]. Many useful compounds have been found and utilized from herbal medicines and natural products to treat various diseases, such as oseltamivir [3] and roscovitine [4]. Oseltamivir is a neuraminidase inhibitor used in the treatment and prophylaxis of both influenza A and influenza B, whereas roscovitine is known as an anticancer drug. However, the process of identification of compound and target protein interactions in vivo and in vitro requires enormous effort. Therefore, efficient in silico screening methods are needed to predict the interaction between compounds and target proteins. In this light, in silico prediction of the interactions between compounds and target proteins can help in making the efforts easier.

As a country with the largest medicinal plant species in the world, Indonesians utilize medicinal plants as a constituent of herbal medicines [5,6,7]. These are known as Indonesian Jamu. Currently, Jamu is produced commercially on an industrial scale under the supervision of the National Agency of Drug and Food Control (NADFC) of Indonesia. Jamu, like the other herbal medicine systems, i.e., traditional Chinese medicine, Japanese Kampo, Ayurveda, and Unani, can be considered as a new resource for compound screening. The molecules might be from a specific part of a plant used as a Jamu ingredient, such as rhizome of Java ginger (*Curcuma xanthorrhiza*), leaf of *kecibeling* (*Strobilanthes crispus*), or fruit of tamarind (*Tamarindus indica*). The utilization of herbal medicines in drug screening is very promising because of the lack of side effects [8,9]. In addition, the high biodiversity in Indonesia has great advantages in the process of finding potential compounds in Jamu. Furthermore, the systematization of Jamu medicine might help not only to obtain information about the major ingredient plants in Jamu medicines, but also to find compound and protein interactions to explain formulation of Jamu. The information on interactions between Jamu compounds and human target proteins will allow understanding the mechanisms of how Jamu medicines work against diseases and will be helpful for finding new drugs based on a scientific basis.

Various screening approaches have been developed to determine candidate compounds from herbal medicines and natural products in drug discovery. One category of the popular approaches is machine learning techniques. This approach can learn from the data, and the resulting model can be applied to make a prediction. Support vector machine (SVM) and random forest are machine learning methods for supervised learning, and they have been used in many research fields with success [10,11,12]. In order to obtain a good model, the machine learning method requires a great number of data samples. Nowadays, there are many open access databases that can be used to support the prediction of compound and protein interactions, such as KEGG [13], DrugBank [14], KNApSAcK [15], UniProt [16], and Online Mendelian Inheritance in Man (OMIM) [17]. Prediction of compound–protein interactions can exploit these databases to identify candidate compounds. In terms of Indonesian Jamu, IJAH Analytics can be considered as a good reference for Jamu because it has information about plant species used in Jamu formulas. In addition, plant species information can be associated with information regarding compounds, target proteins, diseases, and interactions between entities. It is hoped that the more efficient and effective application of natural products will improve the drug discovery process.

Many studies on the prediction of interactions between compounds and target proteins have been reported. Yamanishi et al. implemented a systematic study on the prediction of compound–target protein interactions by utilizing supervised learning using a bipartite graph [18]. The interactions were predicted by utilizing the structural similarity of compounds and the similarity of amino acid sequences. They computed the structural similarities between compounds using SIMCOMP and the sequence similarities between proteins using normalized Smith–Waterman scores [19,20]. In the prediction methods, they applied the bipartite local model (BLM) and SVM to predict compound–target protein interactions [21,22]. BLM predicts target proteins of a given compound using the structural similarity of compounds, proteomic similarity, and information of interactions between compounds and target proteins, whereas SVM was used as the classifier for the BLM.

In this study, we applied machine learning techniques to predict the interaction between compound and protein. SVM and random forest have been chosen as classifiers, and compound and protein are represented by fingerprint and numerical representation of amino acid, respectively. The accuracy, sensitivity, and specificity were used in the evaluation of the models. After we confirmed the best model obtained in the prediction of compound–protein interactions, we determine targeted proteins for candidate compounds obtained from plants used in the Jamu formulas for different efficacies [11]. The objective was not only to identify targeted proteins for developing new drugs, but also to give a comprehensive understanding of Jamu medicines on the molecular level.

## 2. Materials and Methods

**Figure 1:**
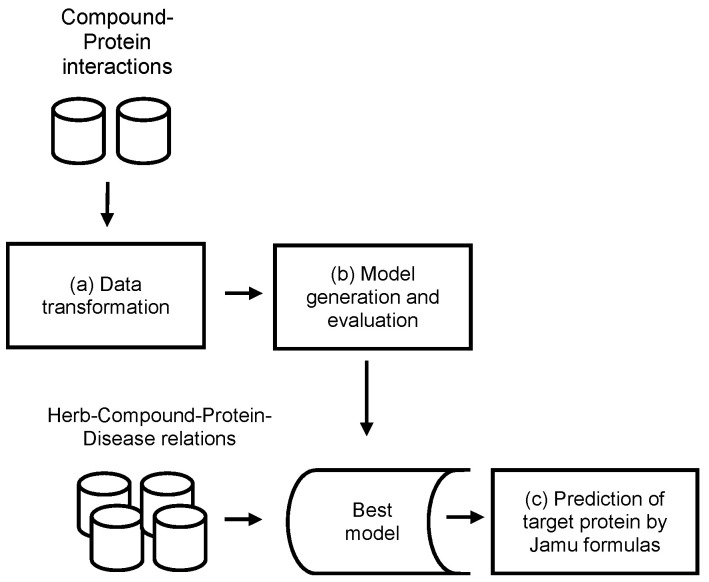
Concept of the methodology.

Jamu medicines consist of a combination of medicinal plants and are used to treat various diseases. In this work, we exploit information about compound and protein interactions from open access databases to predict compound–protein interactions in the context of Jamu formulas. The concept of the proposed method is depicted in Figure 1, which mainly consists of three processes: (a) data transformation, (b) model generation and evaluation, and (c) prediction of targeted proteins by Jamu formulas. 

Initially, we collected the required data for this study from open access databases such as DrugBank, PubChem [23], KNApSAcK, UniProt, KEGG, OMIM, Matador [24], and Indonesian Jamu Herbs (IJAH Analytics, http://ijah.apps.cs.ipb.ac.id, accessed on 20 August 2021). The acquisition of data for generating the prediction model includes compounds, target proteins, and interactions between them. The chemical structures of the compounds were represented by Simplified Molecular Input Line Entry Specification (SMILES) codes. Many databases, such as DrugBank, provide SMILES of each compound [25]. We eliminated some compounds that have ambiguous SMILES or do not have SMILES information. Compounds with known SMILES codes were used in the training process to generate a model for predicting compound–protein interactions. In addition, the information about target proteins was also collected from public databases, especially the IJAH database, and these data were represented by amino acid sequences using FASTA format. In the case of interactions, we gathered that information from IJAH, Matador, and KEGG databases. We also collected the candidate compounds of Jamu formulas associated with efficacy groups from a previous study [11] and used those as test data.

### 2.1. Data Transformation

We transformed information about compounds and amino acid sequences into fingerprints and numerical representations of amino acids, respectively. In the case of compounds, we examined two different fingerprint representations, namely the binary representation of the Molecular Access System (MACCS) and PubChem fingerprints [12,26,27]. Therefore, each compound was represented as 166 and 881 binary vectors, respectively. In the case of proteins, we transformed amino acid sequences into the amino acid composition (AAC) and dipeptide composition descriptors [28]. The AAC represents an amino acid sequence as a fraction of each amino acid type within a protein, and it will produce 20-dimensional AAC vectors. The fractions of all 20 natural amino acids are calculated as:

(1) f(r)=Nr/N, r=1,2,…,20

where $N_{r}$ is the number of the amino acid type *r* and *N* is the length of the sequence. In addition, dipeptide composition will produce 400-dimensional descriptors, defined as:

(2) $$f\left(r,s\right)=\frac{N_{rs}}{N-1},r,s=1,2,\ldots ,20$$

where *N_rs_* is the number of dipeptides represented by amino acid type *r* and type *s*. 

**Figure 2:**
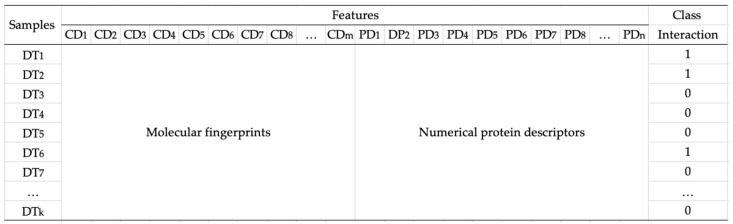
Data representation. Each data sample DT_k_ is composed of molecular fingerprints (CD_1_, CD_2_, CD_3_, …, CD_m_) and numerical protein descriptors (PD_1_, PD_2_, PD_3_, …, PD_n_).

After we transformed compounds and proteins into fingerprints and numerical descriptors, we created four datasets consisting of all combinations of compound and protein vectors for generating the model as follows: a combination of MACCS fingerprint and AACs (called dataset 1), a combination of MACCS fingerprint and dipeptide descriptor (called dataset 2), a combination of PubChem fingerprint and AACs (called dataset 3), and a combination of PubChem fingerprint and dipeptide descriptor (called dataset 4). Figure 2 illustrates the data representation of compounds, proteins, and interactions between them. In the case of testing data, we built combinations of candidate compounds from medicinal plants in Jamu and proteins.

### 2.2. Model Generation and Evaluation

We applied SVM and random forest in the model generation step. SVM is a binary classifier based on constructing an optimal linear model, which has the largest margin between two classes. The linear separator is constructed by simultaneous minimization of the empirical classification error and maximization of the geometric margin [29]. If we have n training data pairs, $T=\left\{\left(x_{i},y_{i}\right)\right\},\;i=1,\ldots ,\;n$, where $x_{i}\left(\in {\mathbb{R}}^{p}\right)$ is a vector representing compound and protein and $y_{i}$ is the class of $x_{i}$. The decision function of SVM is defined as $f\left(x\right)=w^{T}x+b$, where $w={\left[w_{1},w_{2},\ldots ,w_{p}\right]}^{T}$ is the weight vector and b is a scalar. The optimization problem that SVMs aim to minimize is shown in Equation (3):

(3) $$\underset{w\in {\mathbb{R}}^{p},\xi_{i}\in {\mathbb{R}}^{+}}{\min}\frac{1}{2}{\left\|w\right\|}^{2}+C\overset{n}{\underset{i}{\sum }}\xi_{i}$$

subject to $y_{i}\left(w^{T}x_{i}+b\right)\geq 1-\xi_{i}$, where *C* is a trade-off between the width of the margin and the number of misclassifications, and $\xi_{i}$ is a slack variable. SVM can be extended to classify data that are not linearly separable by utilizing a kernel technique. There are two kernel functions that we applied in this study, namely the linear kernel $\left(K\left(x_{i},x_{j}\right)=x_{i}^{T},x_{j}\right)$ and radial basis function (RBF) kernel $\left(K\left(x_{i},x_{j}\right)=e^{-\gamma \left\|x_{i}-x_{j}\right\|{}^{2}},\gamma >0\right)$, where γ is the inverse of the radius of influence of samples selected by the model as support vectors [10,30].

In addition, random forest is an ensemble method composed of many decision trees. For each classification tree, a bootstrap sample of the data is generated, and at each split, the candidate set of variables is a random subset of the variables [31,32,33]. Given a set of training samples $L=\left\{\left(x_{i},y_{i}\right)\right\},i=1,\ldots ,n$, where $x_{i}\left(\in {\mathbb{R}}^{p}\right)$ is a vector of predictor variables representing compound–protein data i and $y_{i}$ is the class label. Random forest targets generating a number of *ntree* decision trees from these samples. The same number of n samples is randomly selected with replacement (bootstrap resampling) for each tree to form a new training set, and the samples not selected are called out-of-bag (OOB) samples. Using this new training set, a decision tree is grown to the largest extent possible without any pruning according to the classification and regression tree (CART) methodology [34]. The Gini index is used during the development process of a decision tree. The Gini index at node v, Gini(v), is shown in Equation (4).

(4) $$Gini\left(v\right)=\overset{C}{\underset{c=1}{\sum }}{\hat{p}}_{c}^{v}\left(1-{\hat{p}}_{c}^{v}\right)$$

where ${\hat{p}}_{c}^{v}$ is the proportion of class c observations at node v [35]. Then, the Gini information gain of $x_{i}$ for splitting node v into two child nodes, $Gain\left(x_{i},v\right)$, is shown in Equation (5):

(5) $$Gain\left(x_{i},v\right)=Gini\left(x_{i},v\right)-w_{L}Gini\left(x_{i}^{L},v^{L}\right)-w_{R}Gini\left(x_{i}^{R},v^{R}\right)$$

where $v^{L}$ and $v^{R}$ are the left and right child nodes of v, $w_{L}$ and $w_{R}$ are the proportions of instances assigned to the left and right child nodes, and $x_{i}^{L}$ and $x_{i}^{R}$ are the instances in the left and right child nodes. At each node, a random set of mtry features out of p is evaluated, and the feature with the maximum $Gain\left(x_{i},v\right)$ is used for splitting the node v. The OOB error is estimated in the process of constructing the forest. After constructing the entire forest, OOB classification results for each sample are used to determine a decision for this sample via a majority-voting rule. 

We defined and compared the performance of the resulting models by using accuracy, sensitivity, and specificity [36,37]. The higher the accuracy is, the better the performance of the classifier is. We measured the accuracies of SVM with two different kernels and random forest using four data representations (datasets 1–4). In order to estimate the performance of random forest and SVM with two different kernels, 10-fold cross-validation was used [21]. Each of the datasets was divided into 10 subsamples. Then, nine samples were used as a training dataset to make a classification model, and the remaining sample was used as a validation dataset for testing the model. In the model evaluation step, we selected the best classifier and data representation of compounds and amino acid sequences for which we obtained the best result and used that for the prediction of target proteins.

### 2.3. Prediction of the Target Protein by Jamu Formulas

**Figure 3:**
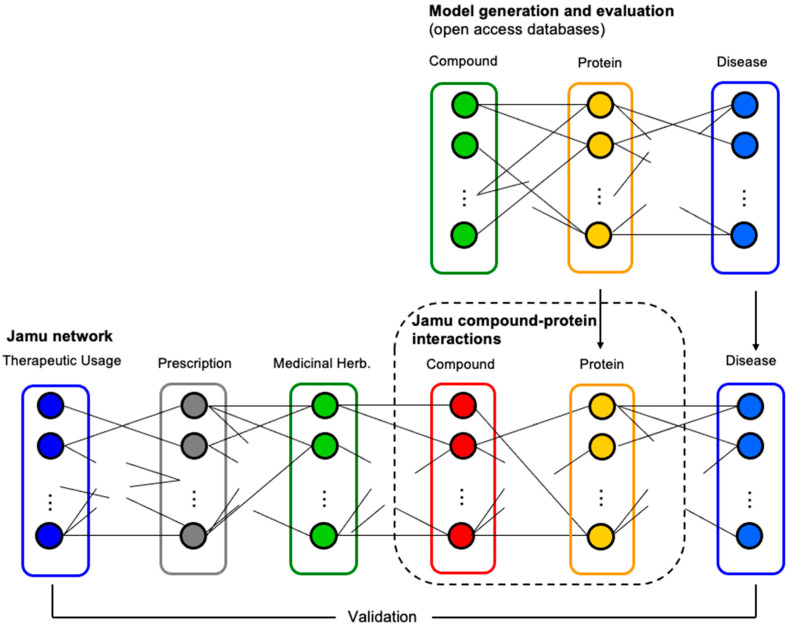
The process of prediction and identification of targeted proteins. Initially, we developed a prediction model of compound–protein interactions by utilizing compound–protein data. Then, the best model was used to predict interaction between compounds of Jamu formulas and their targeted proteins (in the dotted rectangle).

The best model with the highest accuracy was applied for the prediction of compound–protein interactions concerning Jamu formulas used as the testing dataset. In this case, we accepted compound–protein interactions as true interactions when the probability was greater than a threshold. Figure 3 illustrates the relations among different entities involving comprehensive Jamu research, where a dotted rectangle indicates the focus of the present work. Figure 3 also shows how we validate our results by comparing efficacy–compound and protein–disease relations. We validated the results by comparing the therapeutic usage of predicted compounds and the relations of the targeted proteins with diseases. We assessed and discussed the results with supporting evidence from published literature and comments from professional doctors.

## 3. Results and Discussion

The summary of data used in this study is shown in Table 1. We utilized compounds that are reported to be available in the herbs used as Jamu ingredients. There are 17.227 compounds belonging to 4.984 Indonesian herbs collected from KNApSAcK, IJAH, PubChem, and KEGG databases. In addition, the number of target proteins collected from UniProt and IJAH databases is 3.334, and the number of interactions collected from UniProt, IJAH, Matador, and KEGG databases is 7.989. Initially, we removed the data that do not have necessary properties, such as the SMILES in the case of the compounds and the amino sequence in the case of target proteins. Furthermore, we removed the compounds and proteins that are not included in the compound–protein interactions data. We also considered only those compounds that target human proteins. Therefore, the numbers of compounds, proteins, and interactions used in this experiment are 2.146, 3.334, and 7.216, respectively.

### 3.1. Preprocessing of Compound and Protein Data

The transformation of compounds from SMILES to fingerprints was done by utilizing ChemDes web-based software and PaDEL descriptor [27,38]. Compounds were transformed to MACCS and PubChem fingerprints. Moreover, we used the protr package in R to generate AAC and dipeptide as numerical representation schemes of protein sequences [28]. We eliminated two amino acid sequences in the preliminary study, i.e., Q9NZV5 and P36969, because they showed unrecognized amino acid type when transforming amino acid sequences to AAC. Therefore, there were 3.332 proteins left for further processes.

After data transformation finished, we created datasets for compound–protein prediction using both compound and protein space information. Each sample vector is composed of a fingerprint and numerical descriptor of compound and protein. Therefore, for two different compound fingerprints and two protein numerical descriptors, we constructed four matrices with dimensions (2.146 × 3.332) by (166 + 20) for MACCS + AAC, (166 + 400) for MACCS + dipeptide, (881 + 20) for PubChem + AAC, and (881 + 400) for PubChem + dipeptide. The information of interactions between compounds and proteins is considered as a positive class, whereas unknown interactions are considered as a negative class. As the number of samples in the negative class is significantly large (number of compounds multiplies the number of proteins), we randomly selected 7.216 samples for the negative class, the same as the number of positive samples. We determined positive and negative class interactions as classes 1 and 0, respectively.

Wijaya et al. [11] identified 94 significant compounds associated with twelve efficacy groups, and 28 of them were validated by published literature. In this case, the efficacy refers to broad disease classes which are as follows: blood and lymph diseases (E1), cancers (E2), the digestive system (E3), female-specific diseases (E4), the heart and blood vessels (E5), male-specific diseases (E6), muscle and bone (E7), nutritional and metabolic diseases (E8), respiratory diseases (E9), skin and connective tissue (E10), the urinary system (E11), and mental and behavioral disorders (E12). We considered those 94 compounds as test data in this study. Table 2 shows the number of candidate compounds for each efficacy. We transformed the compounds into fingerprints according to the best results we obtained.

### 3.2. Prediction Performance

We applied the R packages named e1071 ver. 1.7–4 to implement the SVM method [39] and randomForest ver.4.6–14 to implement random forest (https://cran.r-project.org/web/packages/randomForest/, accessed on 9 August 2020). The optimal parameters used in the model generations were obtained by utilizing best.tune and tuneRF functions for SVM and random forest, respectively. In the SVM, the regulation parameter *C* depends on numerical protein descriptors. In the case of AACs, *C* is equal to 1, whereas *C* is equal to 1000 in dipeptide. The γ values of datasets 1–4 are 0.00763, 0.00177, 0.00437 and 0.00078, respectively. In random forest, the appropriate number of trees ntree for datasets 1 and 3 is the same, 1000. Additionally, the *ntree* values for datasets 2 and 4 are 2000 and 500, respectively. The mtry values for dataset 2 and 4 are the same, i.e., 10, whereas those for datasets 1 and 2 are 6 and 15, respectively.

Table 3 shows the prediction performance for each type of dataset and each model. Representation of amino acid sequences using AAC descriptor in datasets 1 and dataset 3 obtains better accuracy compared to dipeptide descriptor on both classifiers and compound fingerprints. Furthermore, if we compare the performance of random forest and support vector machine classifiers, the classification accuracy of random forest using AAC descriptor is better than SVM with both kernels. In the case of fingerprints that are used to represent the compounds, MACCS obtains slightly better classification results than PubChem features. One of the reasons for the poor classification results on the dataset using the dipeptide descriptor is the number of features produced by the method. Dipeptide makes 400 features, causing the number of compound–protein features representing the input data to increase. Many features have zero values and affect the resulting model. It is very challenging to determine the most appropriate features because machine learning methods generally rely on feature engineering [40]. This can also be observed in datasets 2 and 4 between MACCS and PubChem fingerprints; when the number of features increases, this also reduces the resulting accuracy. Since this represents sufficiently high performance, the model can be applied to predict interactions between the Jamu compounds and target proteins.

### 3.3. Prediction Results

In order to predict interactions between compounds and target proteins, the classification model was taken from the models that obtained the best classification results. Additionally, a testing dataset was constructed to match the dataset that achieved the best classification result. In this case, we utilized MACCS fingerprint to represent Jamu compounds, AAC descriptor to represent amino acid sequences, and random forest as a classifier. Since we focused on whether compounds bind to target proteins, we created a matrix containing all combinations of candidate compounds of Jamu formulas and target proteins as shown in Figure 2. Then, the prediction model was applied to predict whether compound and protein have an interaction or not. We accepted compound–protein interactions as true interactions when their classification probability was greater than 0.85. Not all candidate compounds identified in the work of Wijaya et al. have interactions with one or more proteins that were utilized in the current experiment. Here, we predicted 168 compound–protein interactions of Jamu formulas, involving 68 candidate compounds. Moreover, the professional doctors validated the predicted compound–protein interactions by comparing the efficacy of predicted compounds and the relations of the targeted proteins with diseases, as shown in Figure 3. Based on the current results, interactions involving 27 compounds can be validated, and those compounds belong to seven efficacy groups. Table 4 summarizes predicted compound–protein interactions by Jamu formulas that have been validated by professional doctors, and all of them are presented under respective efficacies. We also discovered a protein is targeted by many compounds and a compound has interaction with many target proteins. For instance, P02768, known as human serum albumin (HSA), is targeted by caffeic acid, diacetoxy-6-gingerdiol, gallic acid, luteolin, quercitrin, tricin, and ursolic acid. In addition, ursolic acid targets Q92887, Q9NPD5, Q9Y6L6, P08185, and P02768. Further investigation of the predicted compound–protein interactions was also done by finding supporting evidence from published literature, such as HSA being targeted by luteolin [41]. This result indicates that there are some compounds that might be considered as drugs in herbs. This also implies that the prediction model performs well and proteins that are not confirmed yet by any evidence can be candidates to have a relation with the corresponding efficacy group.

## 4. Conclusions and Future Works

We constructed classification–prediction models that predict the interactions between compounds and target proteins using a machine learning approach. The model was created by utilizing compound–protein interaction data obtained from open access databases, and the data were represented by a combination of fingerprint and amino acid sequences. The results showed very good prediction performances, around 90% when the compounds were transformed to MACCS fingerprint, amino acid sequences were transformed to AAC descriptor, and random forest was chosen as a classifier. In addition, some target proteins were predicted from potential compounds of Jamu formulas using the best model obtained in the previous step. By comparing the efficacy of predicted compounds and the relations of the targeted proteins with diseases, we found that some compounds might be considered as drug candidates. There are 27 compounds that can be validated by professional doctors, and those compounds belong to seven efficacy groups. This study is not only determines candidate drugs but also gives a better understanding of Jamu medicine at the omics level. Moreover, further validation of the results of this study can be performed by docking simulation between predicted compound–protein interactions or through in vivo and in vitro validation studies in the laboratory. We can also explore the supporting chemical or biological characteristics in predicted interactions, such as the similarity between the target compound and the known ligands of the predicted protein. 

## Figures and Tables

**Table 1:** The distribution of compound, protein, and interaction between them as training and testing data.

Description	Number of Data	Identifier	References
Protein	3.334	UniProtID	UniProt, IJAH
Compound	17.277	CAS_ID, PubChem ID, KEGG ID	KNApSAcK, PubChem, KEGG, IJAH
Compound of Jamu	94	Compound ID	Wijaya et al. [11]
Compound–protein interactions	149		KEGG
	4.144		Matador
	3.696		UniProt, IJAH
Amino acid sequences	3.334	UniProtID	UniProt

**Table 2:** The number of compounds for predicting target proteins. All data are classified by efficacies, and some compounds are related to one or more efficacy groups.

ID	Efficacy Groups	Number of Compounds
E1	Blood and Lymph Diseases	15
E2	Cancers	5
E3	The Digestive System	17
E4	Female-Specific Diseases	16
E5	The Heart and Blood Vessels	4
E6	Male-Specific Diseases	5
E7	Muscle and Bone	18
E8	Nutritional and Metabolic Diseases	7
E9	Respiratory Diseases	32
E10	Skin and Connective Tissue	4
E11	The Urinary System	14
E12	Mental and Behavioral Disorders	8

**Table 3:** The evaluation of generated models.

Datasets	Classifiers	Accuracy		Sensitivity		Specificity	
MACCS_AAC	SVM Linear	69.16%±	1.07%	71.52%±	1.84%	66.77%±	1.87%
	SVM RBF	81.71%±	1.52%	82.79%±	2.27%	80.62%±	1.27%
	Random Forest	89.30%±	0.69%	87.86%±	1.20%	90.74%±	1.05%
MACCS_Dipeptide	SVM Linear	61.68%±	0.77%	63.49%±	1.61%	61.27%±	0.88%
	SVM RBF	72.71%±	0.86%	71.81%±	1.81%	73.15%±	1.15%
	Random Forest	60.79%±	1.20%	59.14%±	1.56%	61.17%±	1.30%
PubChem_AAC	SVM Linear	70.77%±	0.90%	73.08%±	1.86%	68.49%±	1.87%
	SVM RBF	80.01%±	1.35%	80.52%±	1.80%	79.51%±	1.82%
	Random Forest	89.28%±	0.40%	87.96%±	0.88%	90.63%±	0.58%
PubChem_Dipeptide	SVM Linear	50.49%±	1.08%	54.15%±	1.38%	50.47%±	1.01%
	SVM RBF	49.55%±	1.28%	54.83%±	5.44%	49.56%±	1.19%
	Random Forest	50.28%±	0.72%	50.12%±	1.60%	50.28%±	0.71%

**Table 4:** Predicted compound–protein interactions by Jamu formulas. Compound ID is an identifier taken from PubChem CID (https://pubchem.ncbi.nlm.nih.gov, accessed on 20 August 2021) and KNApSAcK ID (http://kanaya.naist.jp/KNApSAcK_Family/, accessed on 20 August 2021). If the Compound ID cannot be found in PubChem or KNApSAcK databases, we assigned N/A.

No	Compound ID	Compound Name	Molecular Formula	UniProt ID	Targeted Protein	OMIM ID	Disease Description
**E1 Blood and Lymph Diseases**							
1	N/A	(4Z)-1-(2,3,5-Trihydroxy-4-methylphenyl)dec-4-en-1-one	C17H24O4	P02768	Serum albumin	615999; 616000	Hyperthyroxinemia, familial dysalbuminemic; analbuminemia
2	689043, C00000615	Caffeic acid	C9H8O4				
3	5317587,	Diacetoxy-[6]-gingerdiol	C21H32O6				
4	370, C00002647	Gallic acid	C7H6O5				
5	5280445, C00000674	Luteolin	C15H10O6				
6	5280459, C00005373	Quercitrin	C21H20O11				
7	5281702, C00013329	Tricin	C17H14O7				
8	64945, C00003558	Ursolic acid	C30H48O3	Q92887	Canalicular multispecific organic anion transporter 1	237500	Dubin–Johnson syndrome
				Q9NPD5	Solute carrier organic anion transporter family member 1B3	237450	Hyperbilirubinemia, rotor type
				Q9Y6L6	Solute carrier organic anion transporter family member 1B1		Hyperbilirubinemia, rotor type
				P08185	Corticosteroid-binding globulin	611489	Corticosteroid-binding globulin deficiency
				P02768	Serum albumin	615999; 616000	Hyperthyroxinemia, familial dysalbuminemic; analbuminemia
9	73145, C00003738	beta-Amyrin	C30H50O	Q92887	Canalicular multispecific organic anion transporter 1	237500	Dubin–Johnson syndrome
10	222284, C00003672	beta-Sitosterol	C29H50O				Dubin–Johnson syndrome
**E3 The Digestive System**							
1	519857, C00020146	1-epi-Cubenol	C15H26O	P08183	Multidrug resistance protein 1	612244	Inflammatory bowel disease 13
2	N/A	Anisucumarin A	C20H20O8				
3	240, C00034452	Benzaldehyde	C7H6O				
4	6448, C00029844	Bornyl acetate	C12H20O2				
5	3314, C00000619	Eugenol	C10H12O2				
6	289151, C00003162	Longifolene	C15H24				
7	N/A	Morin-3-O-lyxoside	C20H18O11				
8	985, C00001233	Palmitic acid	C16H32O2				
9	442402, C00003194	Thujopsene	C15H24				
10	12306047, C00029671	alpha-Muurolene	C15H24				
11	7460, C00003051	alpha-Phellandrene	C10H16				
12	111037, C00035043	alpha-Terpinyl acetate	C12H20O2				
13	12313020, C00020130	gamma-Muurolene	C15H24				
**E4 Female-Specific Diseases**							
1	5280794, C00003674	Stigmasterol	C29H48O	P11511	Aromatase	139300; 613546	Aromatase excess syndrome; aromatase deficiency
				P03372	Estrogen receptor	615363	Estrogen resistance
**E7 Muscle and Bone**							
1	10131321, C00055009	Coumaperine	C16H19NO2	P20309	Muscarinic acetylcholine receptor M3	100100	Prune belly syndrome
**E8 Nutritional and Metabolic Diseases**							
1	3084331, C00020154	T-Muurolol	C15H26O	Q92887	Canalicular multispecific organic anion transporter 1	237500	Dubin–Johnson syndrome
				Q02318	Sterol 26-hydroxylase, mitochondrial	213700	Cerebrotendinous xanthomatosis
				P11473	Vitamin D3 receptor	277440	Rickets vitamin D-dependent 2A
**E10 Skin and Connective Tissue**							
1	222284, C00003672	beta-Sitosterol	C29H50O	Q02318	Sterol 26-hydroxylase, mitochondrial	213700	Cerebrotendinous xanthomatosis
**E12 Mental and Behavioral Disorders**							
1	6989, C00000155	Thymol	C10H14O	P08172	Muscarinic acetylcholine receptor M2	608516	Major depressive disorder
				Q13002	Glutamate receptor ionotropic, kainate 2	611092	Mental retardation, autosomal recessive 6

## Data Availability

Data used in this study were collected from previous studies and open access databases. Data are available from Computational Systems Biology Laboratory, NAIST, and Department of Computer Science of IPB University for researchers who meet the criteria (contact via correspondence authors).
